# Acute Myocardial Infarction due to External Compression of the Left Main Coronary Artery by a Large Pulmonary Artery Aneurysm

**DOI:** 10.1155/2021/8850044

**Published:** 2021-02-22

**Authors:** H. Sharma, S. N. Doshi, M. A. Nadir

**Affiliations:** ^1^Institute of Cardiovascular Sciences, University of Birmingham, Birmingham, UK; ^2^Cardiology Department, Queen Elizabeth Hospital, Birmingham, UK

## Abstract

**Background:**

Although rare, external compression of the left main coronary artery (LMCA) by a pulmonary arterial aneurysm (PAA) as a consequence of pulmonary arterial hypertension causing stable angina pectoris is well described. However, acute myocardial infarction is extremely rare, particularly with a full array of electrocardiographic, biochemical, and echocardiographic features, as in this scenario.

**Case:**

In this case, a 62-year-old man with a past history of severe fibrotic lung disease was hospitalised with chest pain. The patient had dynamic anterolateral ischaemic changes on electrocardiography and serially elevated high-sensitivity troponin I. Transthoracic echocardiography revealed impaired left ventricular ejection fraction with anterolateral hypokinesis. Coronary angiography with intracoronary imaging revealed external compression of the LMCA. Computer tomography (CT) scans confirmed new PAA, compared to previous scans. The patient was successfully treated by percutaneous coronary stent implantation.

**Conclusion:**

Progressive dilatation of the pulmonary artery due to pulmonary arterial hypertension can result in acute MI secondary to external compression of the LMCA. Clinicians should be mindful of acute coronary syndromes in patients with long-standing pulmonary hypertension presenting with chest pain.

## 1. Introduction

Pulmonary arterial hypertension (PAH) is associated with aneurysm of the main pulmonary artery (PA). In normal anatomy, the PA lies adjacent to the left coronary sinus of the aorta. As the left main coronary artery (LMCA) arises from the left coronary sinus, it may be compressed by a PA aneurysm (PAA). Although rare, a small number of case reports have described this phenomenon in the presence [1, 2] and absence of PAH [3]. The most commonly described clinical sequelae of this scenario is stable angina pectoris [4–7].

Myocardial infarction (MI) as a result of external compression of a coronary artery has been previously documented in patients with anomalous coronary origin or course [8] and normal coronary anatomy [9], attributed to PAA (with or without PAH). However, it is rare to see the full gamut of evidence suggesting acute MI, including dynamic electrogram (ECG) changes, elevation of high-sensitivity troponin, and a corresponding regional wall motion abnormality on echocardiography.

## 2. Case

**Figure 1:**
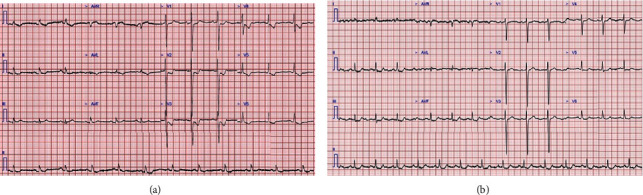
Electrocardiogram demonstrating (a) anterolateral ischaemic changes (ST depression and T wave inversion); (b) older ECG 6 months earlier.

**Figure 2:**
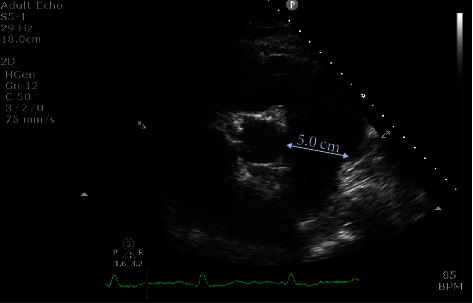
Transthoracic echocardiogram demonstrating significant dilatation of the main pulmonary artery (diameter: 5 cm).

**Figure 3:**
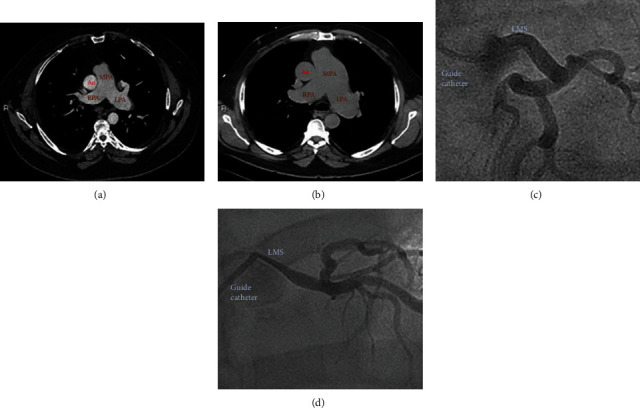
(a) CT scan showing main pulmonary artery with a diameter of 33 mm in 2013; (b) CT scan showing main pulmonary artery with a diameter of 58 mm in 2020; (c) normal appearance of LMCA in 2013; (d) severe tapering stenosis of LMCA in 2020. MPA = main pulmonary artery; RPA = right pulmonary artery; LPA = left pulmonary artery; Ao = ascending aorta; LMS = left main stem.

**Figure 4:**
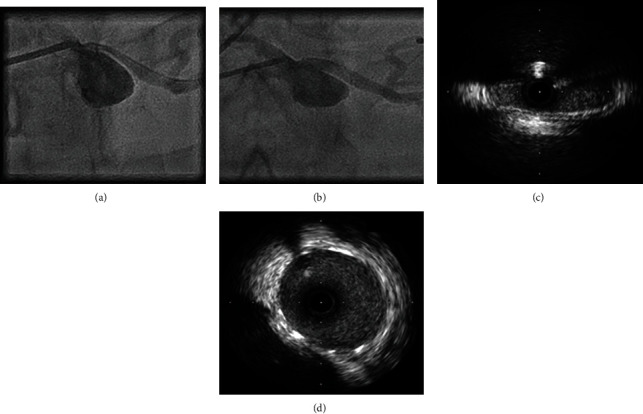
(a) Angiography demonstrating severe ostial LMCA stenosis despite intracoronary nitrate and disengaged guide catheter; (b) angiography following deployment of 5 × 20 mm stent; (c) baseline IVUS with a slit-like lumen of LMCA and distinct lack of atheroma; (d) well-apposed coronary stent and near-circular lumen of LMCA.

A 62-year-old man with a past history of severe fibrotic lung disease requiring long-term oxygen therapy was hospitalised with typical angina pain with no resolution despite sublingual glyceryl trinitrate use. ECG demonstrated dynamic anterolateral ST depression with T wave inversion in anterior and lateral leads (Figure 1). High-sensitivity troponin I assays (normal: < 5 ng/L) measured 99 and 250 ng/L. Transthoracic echocardiography revealed a left ventricle ejection fraction of 52% and anterolateral hypokinesis (video 1) with a high likelihood of pulmonary hypertension (estimated systolic PA pressure 110 mmHg), indicating likely group III pulmonary hypertension (secondary to hypoxic lung disease). The main PA diameter was measured at 5.0 cm (Figure 2). Cardiac catheterisation assessment as part of a lung transplant work-up 7 years earlier had shown no significant coronary disease (Figure 3(c)) with invasive measurements demonstrating only mild pulmonary hypertension (mean systolic PA pressure 30 mmHg). The differential diagnosis included pulmonary thromboembolism and pulmonary/aortic dissection, although the clinical picture was most consistent with acute MI. The patient was commenced on pharmacological treatment for non-ST elevation myocardial infarction. In view of the significant lung disease, an initial conservative approach was taken, but after continual chest pain despite optimal medical therapy, invasive coronary angiography was necessitated. The transradial coronary angiogram showed a tight narrowing of the LMCA ostium (Figure 3(d)). The smooth tapering appearance (Figure 4(a)) raised the possibility of external compression. This was subsequently confirmed with the use of intravascular ultrasound (IVUS) which showed dynamic external compression of LMCA with a slit-like lumen and absence of atheroma (Figure 4(c), video 2)—suggesting that the patient had developed a type 2 NSTEMI due to demand ischaemia. A gated CT scan confirmed normal origin and course of the LMCA; however, it was compressed by a grossly dilated PA measuring 58 mm (normal ~30 mm) at its maximal diameter (Figure 3(b)), compared to 33 mm 7 years ago (Figure 3(a)).

The patient was not felt to be a suitable candidate for surgery and underwent IVUS-guided percutaneous coronary intervention with a 5 × 20 mm everolimus drug-eluting stent deployed directly without any predilatation. Postdeployment, IVUS confirmed a well-apposed stent (video 3) with resolution of extrinsic compression and restoration of the LMCA lumen (Figures 4(b) and 4(d)). The patient was rendered pain free and discharged home after a short period of observation and remained angina free on subsequent follow-up.

## 3. Discussion

MI due to external compression of the left main stem can occur by 2 main mechanisms. In normal coronary anatomy, severe PAA can develop following longstanding PAH, resulting in acute compression of the LMCA. In patients with anomalous coronary origin or course interruption of coronary flow, it occurs due to compression of the LMCA between the PA and the high-pressured aorta and can occur without PAA.

In PAH patients who experience LMCA compression by the PA, stable angina pectoris has been the dominant syndrome reported by numerous case reports, but this case demonstrates that acute MI can also occur, and is supported by electrocardiographic, biochemical, and echocardiographic parameters. The LMCA is the vessel most at risk of compression, and due to the significant myocardial territory supplied, occlusion can result in significant or even life-threatening myocardial injury.

Even if the acute MI event is survivable, major infarction of the LMCA territory in PAH patients could significantly impair long-term prognosis. Such patients are likely to have coexisting right ventricular pressure or volume overload, and acute left ventricular infarction may result in biventricular failure. This has the potential to decompensate pulmonary function due to the combination of pre- and postcapillary pulmonary hypertension. Clinicians should therefore be mindful of acute coronary syndromes occurring in PAH patients presenting with chest pain. As demonstrated in this case, such patients can be easily assessed by CT and intracoronary imaging and successfully treated with prompt percutaneous implantation of an intracoronary stent. It is therefore suggested that extrinsic compression by an aneurysmally dilated PA should be considered in the differential diagnosis of chest pain in patients with severe PAH. Coronary angiography alone can misdiagnose extrinsic LMCA compression, but IVUS can more accurately determine the extent of LMCA stenosis and is associated with lower rates of major clinical events [10].

It is worth noting that because of the slit-like nature of the narrowing, 45° left anterior oblique (LAO) or 30° LAO cranial angulation coronary angiographic planes cross-sectioning the narrow axis of the compressed LMCA best visualise this pathology, whereas other planes crossing the wide axis of the narrowing frequently miss the compression [11]. Shorter stents are often necessary to eliminate the risk of circumflex artery obliteration. Moreover, displacement of the aortic lumina is an important risk during ostial LMCA stenting. Intravascular ultrasound imaging can aid diagnosis (absence of plaque and dynamic external compression) and determine stent size and optimal position of deployment.

## Data Availability

Data are available on request.

## Additional Points

*Learning Objectives*. Longstanding PAH may result in PAA. Patients with known PAA and chest pain should be considered for urgent coronary angiography with IVUS imaging to diagnose and treat possible extrinsic coronary compression. 45° left anterior oblique (LAO) or 30° LAO cranial coronary views are best to visualise the narrowing (other views frequently miss the compression). IVUS can aid diagnosis and determine stent size and optimal position of deployment.

## Supplementary Materials

Supplementary Materials Video 1: transthoracic echocardiography demonstrating anterolateral left ventricular hypokinesis. Video 2: intravascular ultrasound examination of the left main coronary artery, demonstrating external compression with a slit-like lumen and absence of atheroma. Video 3: intravascular ultrasound examination of the left main coronary artery, demonstrating resolution of external compression and well-apposed stent.
